# Supplementary material for: Overexpression of the Glycyrrhiza uralensis Phenylalanine Ammonia-Lyase Gene GuPAL1 Promotes Flavonoid Accumulation in Arabidopsis thaliana
Source: Int J Mol Sci. 2025 Apr 25;26(9):4073. doi: 10.3390/ijms26094073 (PMC12071455; doi:10.3390/ijms26094073)
Supplement: Supplementary file 1 [file ijms-26-04073-s001.zip › ijms-3567624-supplementary/Supplementary Figure S1-S2.pdf]

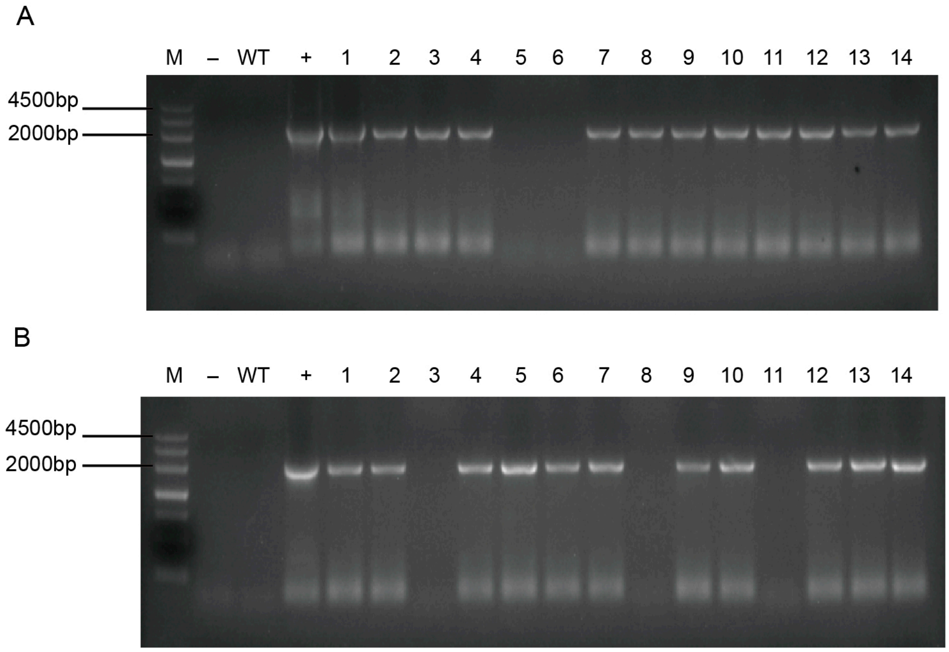

Figure S1. PCR identification of the *GuPAL1* gene in transgenic *Arabidopsis thaliana* positive plants. (A) Molecular verification of *GuPAL1*-OE *Arabidopsis thaliana* lines. Fourteen putative *GuPAL1*-OE transgenic lines were genotyped by PCR amplification using *GuPAL1*-specific primers, followed by 1% agarose gel electrophoresis analysis. Twelve transgenic lines (excluding samples 5 and 6) exhibited the expected amplification fragment corresponding to the positive control (+), confirming successful *GuPAL1* overexpression. (B) Molecular verification of *Atpal1*/*GuPAL1* complementation lines. Fourteen candidate complementation lines were similarly analyzed using *GuPAL1*-specific primers. Eleven lines (excluding samples 3, 8 and 11) showed the correct PCR product matching the positive control, demonstrating successful complementation of the *atpal1* mutant with *GuPAL1*.

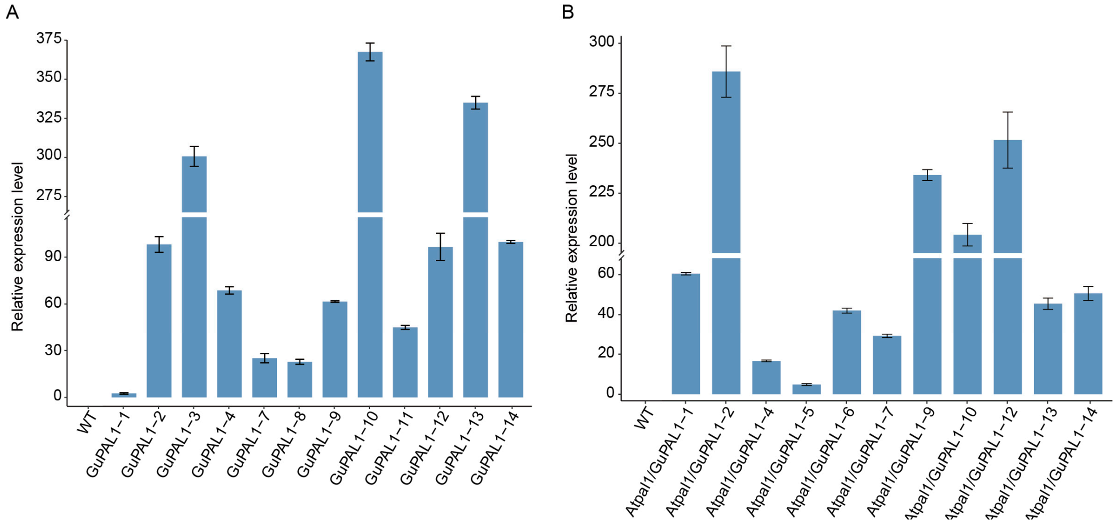

Figure S2. qRT-PCR expression analysis of *GuPAL1* in transgenic *Arabidopsis thaliana* positive lines. (A) Twelve transgenic *Arabidopsis thaliana GuPAL1*-OE that tested positive in DNA identification were subjected to qRT-PCR analysis. Based on the results, three lines (*GuPAL1-3*, *GuPAL1-10,* and *GuPAL1-13*) exhibiting high expression levels were selected for subsequent experiments. (B) Eleven DNA-positive *Arabidopsis thaliana* lines with functional complementation of *Atpal1* were analyzed by qRT-PCR. Four lines (*Atpal1*/*GuPAL1*-2, *Atpal1*/*GuPAL1*-9, *Atpal1*/*GuPAL1*-10, and *Atpal1*/*GuPAL1*-12) showing high expression were chosen for further studies.
